# Clinical Outcomes Following a Change in Gestational Diabetes Mellitus Diagnostic Criteria Due to the COVID-19 Pandemic: A Case-Control Study

**DOI:** 10.3390/ijerph19031884

**Published:** 2022-02-08

**Authors:** Niamh Keating, Kirsten Carpenter, Kelsey McCarthy, Ciara Coveney, Fionnuala McAuliffe, Rhona Mahony, Jennifer Walsh, Mensud Hatunic, Mary Higgins

**Affiliations:** 1 UCD Perinatal Research Centre, National Maternity Hospital, University College Dublin, 4 Dublin, Ireland; niamh.keating@ucd.ie (N.K.); fionnuala.mcauliffe@ucd.ie (F.M.); jwalsh@nmh.ie (J.W.); 2 UCD School of Medicine, 4 Dublin, Ireland; kirsten.carpenter@ucdconnect.ie (K.C.); kelseymccarthy@gmail.com (K.M.); 3 Department of Midwifery, National Maternity Hospital, 2 Dublin, Ireland; ciara.coveney@nmh.ie; 4 Obstetrics and Gynaecology, National Maternity Hospital, 2 Dublin, Ireland; rmahony@nmh.ie; 5 Department of Endocrinology, Mater Misericordiae Hospital, 7 Dublin, Ireland; mhatunic@mater.ie

**Keywords:** COVID-19, gestational diabetes mellitus, antenatal care

## Abstract

Background: Due to COVID-19, many centres adopted a change to the diagnosis of GDM. Methods: A case-control study of antenatal patients between 1 April and 30 June in 2019 and 2020 looking at detection rates of GDM, use of medication, obstetric, and fetal outcomes. Results: During COVID-19, the rate of positive GDM tests approximately halved (20% (42/210) in 2020 vs. 42.2% (92/218) in 2019, (*p* < 0.01)) with higher rates of requirement for insulin at diagnosis (21.4% (2020) vs. 2.2% (2019); *p* < 0.01), and at term (31% (2020) vs. 5.4% (2019); *p* < 0.01). and metformin at diagnosis (4.8% (2020) vs. 1.1% (2019); *p* < 0.01), and at term (14.3% (2020) vs. 7.6% (2019) *p* < 0.01), with no differences in birth outcomes. Conclusions: There was likely an underdiagnosis of GDM while women at a higher risk of hyperglycaemia were correctly identified. The GTT should be maintained as the gold-standard test where possible, with provisions made for social distancing during testing if required.

## 1. Introduction

Gestational diabetes mellitus (GDM) is defined as hyperglycaemia first detected during pregnancy [1] and affects approximately one in six pregnancies [2]. It is associated with an increased risk in neonatal and obstetric morbidity [3] and is strongly predictive of later type 2 diabetes mellitus in affected women [4]. A diagnosis of GDM is therefore important, not only in improving birth outcomes by improving glycaemic control, but also in identifying at-risk women so that they may adopt strategies such as diet and exercise to reduce their risk or delay progression to future T2DM. Untreated, GDM is associated with increased neonatal morbidity [5].

During the COVID-19 pandemic, significant changes were made to the provision of maternity services. Unlike many elective hospital services that were temporarily suspended, maternity care could not be cancelled but had to be modified. “Social distancing” is a cornerstone of limiting the spread of COVID-19 [6]. Changes made to maternity care included adaptations in order to limit the number of women attending the hospital and the time spent there, thereby reducing the risk of women contracting the virus through hospital contact.

The oral glucose tolerance test (GTT) is considered the gold-standard in the diagnosis of GDM. There is much debate over the merits of a one-step (GTT) or two-step (glucose challenge test (GCT) for screening, and if positive, then GTT for diagnosis) approach for diagnosis, and this was the focus of a recent randomised control trial [7] that showed no difference in outcomes between the two testing strategies.

During the pandemic, there were concerns about having women spend three hours in the hospital during a busy period with limited waiting room capacity. Recommendations from the Royal College of Obstetricians and Gynaecologists, published in March 2020 [8], suggested changing the diagnostic test to fasting plasma glucose and HbA1c. A diagnosis of GDM was made if a fasting glucose level was ≥5.3 mmol/L or a HbA1C of ≥39 mmol/mol. A similar approach of replacing the OGTT with alternative testing has been adopted in countries such as Australia [9] and Canada [10]. In Ireland, the Institute for Obstetrics and Gynaecology issued guidance for the management of women with GDM but did not specify a diagnostic approach [11]. These recommendations were intended to be temporary during the COVID-19 pandemic.

The aim of this study was to compare the incidence of GDM and obstetric and neonatal outcomes during the first wave of the COVID-19 pandemic with the previous year.

## 2. Materials and Methods

This was a retrospective case-control study carried out in a tertiary Dublin hospital with approximately 9000 births per year where approximately 900 women are diagnosed with gestational diabetes each year. Women diagnosed with GDM are managed in a multidisciplinary service, including obstetrics/maternal-fetal medicine, endocrinology, midwifery, specialist midwifery and diabetes, dietetics, and social work, with support of other specialties if required.

The normal practice of diagnosing GDM in the National Maternity Hospital is a two-step approach where women with identifiable risk factors undergo glucose challenge testing (GCT) [12]. Risk factors include a body mass index (BMI) > 30, maternal age > 40, family history of diabetes, previous unexplained perinatal death, previous macrosomia of baby > 4.5 kg, and ethnicity associated with a high prevalence of diabetes. The glucose challenge test (GCT) takes the form of glucose level taken one hour after a 50 g carbohydrate load. A screen-positive result is a glucose level greater than or equal to 7.8 mmol/L. Those identified as being screen-positive then undergo formal testing in the form of an oral glucose tolerance test (GTT), carried out over three hours. In our unit this is performed with a 100 g carbohydrate load. Normal results are fasting <5.3 mmol/L, one hour <10.0 mmol/L, 2 h < 8.6 mmol/L, and 3 h less than 7.8 mmol/L. A diagnosis of GDM is made if 2 or more out of 4 abnormal values are detected.

Due to concerns about performing the GTT as outlined previously, changes to our diagnostic procedure were introduced on 1st April 2020. We do not routinely test at-risk women at booking and continued to use the GCT as a first step for at-risk women between 24 and 28 weeks. Changes introduced meant that those with a positive GCT underwent a single fasting plasma glucose test and HbA1C. In July 2020, we reverted to our previous screening strategy.

Pregnant women with a positive GCT from 1st April to 30th June 2019, and 1st April to 30th June 2020 were identified from hospital records and confirmed by the laboratory database. This timeframe was chosen as it represents the period of change in the diagnosis of GDM during the first wave of the pandemic. Following this we reverted to standard testing with social distancing measures. Only women with no prior history of GDM underwent screening as per local policy and therefore none of the women included in this study had been diagnosed with GDM in a previous pregnancy.

Ethical approval was granted by the Research Ethics Committee at the National Maternity Hospital (RA.20.2021). De-identified data on patient demographics, indication for GCT, results of GCT, subsequent testing, obstetric care and outcomes, and neonatal outcomes were recorded from the hospital electronic chart by two researchers to reduce the possibility of error.

Patients were divided into the following four groups: those with a positive GTT in 2019, those with a negative GTT in 2019 (prior to the pandemic induced change in screening practice), those with a “positive” diagnostic test in 2020 (i.e., raised fasting glucose and/or HbA1c), and those with a “negative” diagnostic test in 2020. Outcomes in each group were compared based on obstetric (fetal growth centiles, requirement for induction of labour, mode of birth, gestational age at birth, and estimated maternal blood loss) and neonatal outcomes (birth weight, rate of shoulder dystocia, rate of neonatal hypoglycaemia, requirement for admission to neonatal unit, and neonatal head circumference and length centiles) [13].

Analysis was performed with IBM SPSS Statistics version 26. Categorical variables were compared with a Chi-squared test with independent t-test performed for parametric data and Mann–Whitney U test for non-parametric data. As this was exploratory research of a convenience sample, power calculations were not possible and therefore the *p* value of <0.05 indicates an impression of statistical significance.

## 3. Results

The total number of live births was 2004 from 1st April to 30th June 2019 and 1886 in the three-month period in 2020. There were 974 GCTs performed in 2019 during the study period and 937 performed during the same period in 2020 (*p* < 0.05). Between April and June 2019, 218 women with a singleton pregnancy had a positive GCT; in the same period in 2020, 210 women with a singleton pregnancy had a positive GCT (*p* < 0.05).

Three women did not undergo the second-step GTT in 2019 following positive GCT late in the third trimester for clinical suspicion of macrosomia. All three women delivered within one week of a positive screening test. One woman did not undergo diagnostic testing in 2020 for clinical suspicion of macrosomia following a positive GCT; she appears to have been lost to follow-up. She went on to deliver a 4.1 kg baby at 41 weeks’ gestation, and the infant was admitted to the NICU for management of hypoglycaemia. Of note, three women had abnormal fasting glucose or HbA1C but were not linked with the diabetes team. For the purpose of analysis, these women were included as having GDM. None of the women recruited tested positive for COVID-19 during the study period.

Demographic information for the total group and the separate four groups is shown in Table 1, with birth information in Table 2.

There was a substantial decrease in the rate of diagnosis of GDM as a result of the change in diagnostic criteria, from 42.2% (92/218) in 2019 to 20% (42/210) in 2020 (*p* ≤ 0.01). This roughly equates to an incidence of GDM of 4.9% (92/2004 births) in 2019 and 2.2% (42/1886 births) in 2020.

There were no differences in the demographics of patients in each cohort of 2019 and 2020 based on age, body mass index (BMI), and nulliparity. There was no difference in birth outcomes for each group (Supplementary Materials, Table S1). Women with a negative test in 2019 and 2020 were compared, with no difference in outcomes (Supplementary Materials, Table S2).

Babies of women who tested negative for GDM in 2020 (Table 3) had an increase in head circumference compared with those testing positive for GDM (75th centile vs. 61st centile, *p* = 0.014) and length centiles (79th centile vs. 50th centile, *p* = 0.021), but this was not reflected in absolute birth weight (3680 g in the GDM negative group vs. 3488 g in the GDM positive group, *p* = 0.153) or birth weight centiles > 90th (27.5% in the GDM negative group vs. 19% in the GDM positive group, *p* = 0.261). There was one shoulder dystocia in the 2020 cohort and one in 2019, both in women without GDM (i.e., positive GCT but negative diagnostic test).

There was a significant increase in post-partum haemorrhage (defined as blood loss >500 mL) in the women who were negative for GDM compared with those who were positive in 2020 (11.9% vs. 30.5%, *p* = 0.015) (Table 3).

Repeat testing based on clinical suspicion was performed in 15 women in 2019 and 22 women in 2020, with seven women subsequently diagnosed with GDM in 2019 and 10 women in 2020, a difference that was not significant (*p* = 0.942). Of these 10 women in 2020, six were diagnosed on repeat testing with standard GTT and two had home testing with glucometers based on clinical suspicion of gestational diabetes. Both of these women required insulin from a diagnosis of GDM in the third trimester.

Following diagnosis, early recourse to insulin and metformin was more likely in 2020, as shown in Table 4. Insulin was initiated at diagnosis in 21.4% of women in 2020 compared with 2.2% in 2019 (*p* < 0.01). Initiation of metformin at diagnosis was 4.8% in 2020 compared with 1.1% in 2019 (*p* < 0.01). There was also an increase in the use of insulin and metformin at birth. At term, 31% of women required insulin to control GDM in 2020, compared to 5.4% of women with GDM in 2019 (*p* < 0.001). For treatment at term the rates for Metformin were 14.3% in 2020, compared with 7.6% in 2019 (*p* < 0.001). This increase in medication use did not result in an increase in birth weight, induction of labour, Caesarean or instrumental birth rates (Table 4).

## 4. Discussion

In our cohort of women with a positive GCT, there was a reduction in the rate of GDM based on a change in diagnostic criteria during the COVID-19 pandemic. There was an increased requirement for insulin or metformin treatment for GDM, but no differences in other maternal, fetal, or neonatal outcomes other than a difference in the rate of PPH. There was no difference in risk factors for PPH between the two groups or in the PPH rate for all deliveries in that period, so we conclude this may be a Type 2 error.

The diagnosis of GDM during the pandemic has been identified as a challenge worldwide, with different units having to adapt testing to meet the recommendations for social distancing and mitigate against the potential exposure of pregnant women to COVID-19. A review of proposed protocols in the United Kingdom, Canada, and Australia, using theoretical modelling was performed [14]. This study used a secondary analysis from the HAPO cohort and applied different diagnostic criteria to explore outcomes. In keeping with our study, diagnostic criteria during the pandemic showed a reduction in the frequency of diagnosed GDM without an increased risk in obstetric or neonatal complications of GDM (13). The reduction in GDM was theoretically highest in the UK (81%) and Canada (82%), while the Australian criteria showed a potential 25% reduction.

To our knowledge, one other study has been published on their experience following the change in diagnostic criteria. In a single-centre observational study in London, researchers showed that the introduction of RCOG COVID-19 gestational diabetes screening criteria failed to detect 57% of women subsequently identified as GDM on testing with OGTT [15]. Clinical differences in that study compared to ours are that they screened for GDM at booking and then again at 28 weeks using a one-step GTT. They have not yet reported on pregnancy outcomes or need for treatment, and we eagerly await their long-term follow-up for comparison with our findings.

A study looking at the alternative criteria for diagnosis of GDM in Queensland, Australia, identified a fasting blood glucose level of >4.6 mmol/L as being the optimal predictor for elevated post-glucose load blood glucose levels [16]. The specificity of this was 77%, but the sensitivity was only 54%. Applying this cut-off of FBG > 4.6 mmol/L, the positive cases in 2020 in our cohort would be 42.4% (n = 89), which would be in line with the detection rates of GDM following positive screening with GCT in the previous year. An analysis of fasting glucose levels over a six-year period in Australia demonstrated that this same cut-off of 4.6 mmol/L would miss gestational diabetes in a third of women who otherwise would have been diagnosed [17]. Clearly, there is a balance to be met. A diagnosis of diabetes in pregnancy means more intensive monitoring of mother and fetus with additional education sessions, glucose monitoring, and fetal ultrasound, all of which have the potential to increase potential COVID-19 exposure through additional travel and hospital contact. Women have reported that the diagnosis is shocking and traumatic, though these emotions tend to improve as women move through the pregnancy and adjust to the diagnosis [18]. As with other studies, we observed a trade off in specificity over sensitivity in diabetes diagnosis [19]. From July 2020, the RCOG has advised that the suggested modifications to GDM screening apply to the peak of the pandemic and services should return to previous strategies as soon as local prevalence and risk allow. During the subsequent second and third peaks observed in Ireland, we continued to use our standard diagnostic process with emphasis on social distancing, hand hygiene, and mask-wearing in waiting areas, with no cases of COVID-19 transmission related to GTT appointments.

To our knowledge, this is the first study to look at the clinical outcomes following a change in diagnostic criteria during COVID-19 pandemic. A limitation of this study is the lack of generalisability of results given the different strategies adopted by different countries and, indeed, differences between units within Ireland. Further research is needed to compare different strategies for diagnosing gestational diabetes in the event of future epidemics/pandemics, although given the variation in GDM screening that already exists, it is unlikely a “one-size-fits-all” approach as an alternative to the GTT will be found. It is recognized that HbA1C has a limited role in pregnancy due to an increased turnover of red blood cells and that it can underestimate glucose intolerance, particularly in women with anaemia [20].

One potential confounding factor during COVID-19 is the change in lifestyle associated with lockdown, which saw people change their daily routine, with more people work from home. This may account for the increase in medication use for the management of GDM during this period, although as mentioned previously, the tests that were implemented were poorly sensitive while being highly specific, which is likely to have contributed to increased medication use.

## 5. Conclusions

Replacing the gold-standard of GTT with fasting glucose and HbA1C risks underdiagnosing GDM. This may have potential implications for future pregnancies and long-term health. The change in the diagnostic criteria of GDM was a pragmatic decision made in the face of unprecedented challenges in maternity service provision. While adverse perinatal outcomes were not increased in this cohort, the numbers were small. Our higher rates of medication use (insulin and metformin) in 2020 would signify that women who met criteria for diagnosis of GDM using the criteria during the pandemic had a higher degree of hyperglycaemia, or in essence, women with mild hyperglycaemia who would have ultimately been diet controlled did not fulfil the criteria in this screening process. A diagnosis of GDM offers an opportunity to identify women at an increased risk of type 2 diabetes in the medium- and long-term and can impact future obstetric care and future health risk stratification and screening. It is important that this opportunity is not lost, and that future pandemic care should maintain the use of the GTT as the gold-standard test where possible, and every attempt should be made to make provisions for social distancing for women who are undergoing testing. Adverse perinatal outcomes are associated with the hyperglycaemic patients with GDM, not those with well-controlled GDM. It would appear the change in approach during the COVID-19 pandemic correctly identified those at risk of poor obstetric and neonatal outcomes.

## Figures and Tables

**Table 1:** Demographic information for patients with a positive glucose challenge test from 1st April to 30th June 2019 (routine screening for GDM using a two-step approach with GCT and glucose tolerance test) and 1st April to 30th June 2020 (modified screening for GDM due to the COVID-19 pandemic).

	Positive GCT 2019 N = 218	Positive GTT 2019 n = 92	Negative GTT 2019 n = 123	Positive GCT 2020 n = 210	Positive HbA1c or Fasting Glucose 2020 n = 42	Negative HbA1c and Glucose 2020 n = 167
Maternal Age (mean/range)	36 (20–49)	36 (20–44)	36 (22–49)	35 (20–47)	34 (24–45)	36 (20–47)
Maternal Primiparity (n and % primiparous women)	96 (44%)	47 (51.1%)	49 (39.8%)	102 (48.6%)	26 (61.9%)	56 (33.5%)
Maternal BMI (mean/range)	26.7 (17–45.3)	28.5 (19.9–43.1)	25.4 (17–45.3)	27.3 (18.3–51.4)	29.4 (22.59–51.39)	26.93 (18.34–44.8)
Maternal Ethnicity, n (%)						
White Irish	141 (64.7%)	63 (68.5%)	76 (61.8%)	115 (54.8%)	13 (31%)	102 (61%)
Irish Traveller	0	0	0	2 (1%)—no diagnostic test = 1	0	1 (0.6)
Other white background	41 (18.8%)	12 (13%)	29 (23.6%)	42 (20%)	7 (16.7%)	35 (21%)
Asian or Asian Irish	28 (12.8%)	13 (14.1%)	14 (11.4%)	40 (19%)	18 (42.9%)	22 (13.2%)
Black or Black Irish	1 (0.5%)	0	1 (0.8%)	7 (3.3%)	3 (7.1%)	4 (2.4%)
Other including mixed backgrounds	3 (1.4%)	2 (2.2%)	1 (0.8%)	3 (1.4%)	1 (2.4%)	2 (1.2%)
Not recorded	4 (1.8%)	2 (2.2%)	2 (1.6%)	1 (0.5%)		
Maternal gestation at GCT, mean (range)	28 (8–40)	29 (14.4–38)	28 (8–39)	28 (11.3–38)	28 (11.3–38)	28 (12–39)
Maternal GCT result, mean (SD)	8.6 (0.1)	9 (1.2)	8.4 (0.9)	9.15 (1.7)	9.15 (1.7)	8.5 (10)
Abnormal fasting glucose					25 (59.5%)	
Abnormal HbA1C					20 (47.6%)	
Both diagnostic tests abnormal					11 (26.2%)	
Initial treatment, n (%)						
Diet and exercise only		89 (96.7%)			28 (66.7%)	
Metformin		1 (1.1%)			2 (4.8%)	
Insulin		2 (2.2%)			9 (21.4%)	
Term treatment, n (%)						
Diet and exercise only		79 (85.9%)			19 (45.2%)	
Metformin		7 (7.6%)			6 (14.3%)	
Insulin		5 (5.4%)			13 (31%)	
Insulin and metformin		1 (1.1%)				

BMI: body mass index; GCT: glucose challenge test; GTT: glucose tolerance test.

**Table 2:** Birth information for infants of pregnant patients with a positive glucose challenge test from 1st April to 30th June 2019 (routine screening for GDM using a two-step approach with GCT and glucose tolerance test) and 1st April to 30th June 2020 (modified screening for GDM due to the COVID-19 pandemic).

	Positive GCT 2019 n = 218	Positive GTT 2019 n = 98	Negative GTT 2019 n = 131	Positive GCT 2020 n = 210	Positive HbA1c or Fasting Glucose 2020 n = 42	Negative HbA1c and Glucose 2020 n = 167
GA at birth (mean/range)	39.6 (33.3–42.14)	39.6 (33.7–41.7)	39.7 (33.3–42.14)	39.5 (26.4–42.1)	39.3 (32.6–41.3)	39.7 (26.4–42.1)
Induction of labour, n (%)	64 (29.4%)	31 (33.7%)	32 (26%)	79 (38%)	20 (47.6%)	58 (34.7%)
Mode of Delivery, n (%)						
Spontaneous vaginal delivery	110 (50.5%)	47 (51.1%)	62 (50.4%)	101 (485)	19 (45.2%)	81 (48.5%)
Instrumental delivery	24 (11%)	13 (14.1%)	11 (8.9%)	25 (12%)	3 (7.1%)	22 (13.2%)
Caesarean section	84 (38.5%)	32 (34.8%)	50 (40.7%)	84 (40%)	20 (47.6%)	65 (39%)
Birth weight (g), range	3633 (2070–4920)	3607 (2530–4630)	3650 (2070–4920)	3648 (975–4880)	3488 (1960–4880)	3680 (975–4850)
>90th centile, n (%)	43 (19.7%)	17 (18.5%)	25 (20.3%)	55 (26.2%)	8 (19%)	46 (27.5%)
PPH > 500 mL, n (%)	46 (21.3%)	19 (20.7%)	26 (21.3%)	56 (26.7%)	5 (11.9%)	51 (30.5%)
Shoulder dystocia, n (%)	1 (0.5%)	0	1 (0.8%)	1	0	1
NICU admission, n (%)	57 (26.3%)	28 (30.4%)	29 (23.8%)	50 (23.8%)	11 (26.2%)	39 (23.4%)
Hypoglycaemia	13 (6%)	7 (7.6%)	6 (4.9%)	6 (2.9%)	4 (9.5%)	2 (1.2%)
Jaundice	5 (2.3%)	2 (2.2%)	3 (2.4%)	11 (5.2%)	1 (2.4%)	10 (6%)
Antibiotics	0	0	0	2 (1%)	1 (2.4%)	1 (0.6%)
TTN	8 (3.7%)	3 (3.3%)	5 (4.1%)	5 (2.4%)	0	5 (3%)
Other	26 (11.9%)	13 (14.1%)	13 (10.6%)	24 (11.4%)	6 (14.3%)	18 (10.8%)
Head circumference centile, mean	70.3	64	71.8	75	61	75
Length centile, mean	74.8	73.7	75	75	50	79.1

**Table 3:** Comparison of outcomes from pregnancies diagnosed with gestational diabetes (GDM) in 2019 (routine screening for GDM using a two-step approach with GCT and glucose tolerance test) and 2020 (modified screening for GDM due to the COVID-19 pandemic).

	All GDM Positive 2019 n = 92	All GDM Positive 2020 n = 42	*p*-Value
BMI, mean( SD)	28.5 (5.6)	29.4 (6.6)	0.15
Age, mean (SD)	36 (4.9)	34.(5.7)	0.1
Gestational age at glucose Challenge test (weeks), mean (SD)	29 (4.1)	28 (5)	0.04
Booking weight, mean (SD)	74.7 (16)	74.7 (17.8)	0.52
Infant head circumference (cm), mean (SD)	64 (29.4)	61 (32)	0.37
Infant length (cm), mean (SD)	73.7 (26.3)	50 (31)	0.08
Birth weight (g), mean (SD)	3608 (481)	3488 (638)	0.44
Rate of postpartum Haemorrhage (PPH), n (%)	19 (20.7%)	5 (11.9%)	0.21
Birth weight > 90th centile, n (%)	17 (18.5%)	8 (19%)	0.96
Gestational age at birth (weeks), mean (SD)	39.6 (1.3)	39.3 (1.5)	0.06
Induction of labour, n (%)	31 (33.7%)	29 (47.6%)	0.06
Mode of birth, n (%)			
Spontaneous vaginal birth	47(51.1%)	19 (45.2%)	0.7
Instrumental birth	13 (14.1%)	3 (7.1%)	0.8
Caesarean section	32 (34.8%)	20 (47.6%)	0.16
Initial treatment of GDM			<0.01
Diet and exercise only	89 (96.7%)	31 (73.8%)	
Metformin	1 (1.1%)	2 (4.8%)	
Insulin	2 (2.2%)	9 (21.4%)	
Term treatment			<0.01
Diet and exercise alone	79 (86%)	21 (50%)	
Metformin	7 (7.6%)	6 (14.3%)	
Insulin	5 (5.4%)	13 (31%)	

BMI: body mass index, GA: gestational age, GCT: glucose challenge test, GDM: gestational diabetes.

**Table 4:** Comparing those with a positive diagnostic test for GDM in 2020, with those who had tested negative for GDM following a screen-positive GCT result.

	GDM pos 2020 n = 42	GDM neg 2020 n = 167	*p*-Value
BMI (mean, SD)	29.4 (6.6)	26.9 (5.7)	0.06
Age, years (SD)	34.(5.7)	36 (4.9)	0.13
GA at GCT	28 (5)	28 (4.1)	0.54
Booking weight (mean, SD)	74.7 (17.8)	72.2 (16)	0.1
Infant head circumference centile (mean, SD)	61 (32)	75 (26.2)	0.01
Infant length centile (mean, SD)	50 (31)	79.1 (28.8)	0.02
Birth weight, weight in grams (mean, SD)	3488 (638)	3680 (570)	0.15
PPH (>500 mL), n (%)	5 (11.9%)	51 (30.5%)	0.02
Birth weight > 90th centile, n (%)	8 (19%)	46 (27.5%)	0.26
Gestational age at birth (weeks) mean, SD)	39.3 (1.5)	39.7 (1.9)	0.08
Induction of labour, n (%)	20 (47.6%)	58 (34.7%)	0.03
Mode of delivery			
SVD, n (%)	19 (45.2%)	81 (44.3%)	0.54
Instrumental birth, n (%)	3 (7.1%)	22 (37.4%)	0.28
Caesarean birth, n (%)	20 (47.6%)	64 (38.3%)	0.54
NICU admission, n (%)	10 (23.8%)	39 (23.4%)	0.95

GA: gestational age, GCT: glucose challenge test, NICU: neonatal intensive care, PPH: postpartum haemorrhage, SVD: spontaneous vaginal delivery.

## Data Availability

The data of this study are available upon reasonable request.

## Supplementary Materials

The following supporting information can be downloaded at: https://www.mdpi.com/article/10.3390/ijerph19031884/s1, Table S1. Maternal and neonatal outcomes compared based on those with a positive Glucose Challenge Test (GCT) in 2019 to those with a positive GCT in 2020; Table S2. Comparing all those with a positive Glucose Challenge Test (GCT) but negative second step diagnostic test (Glucose Tolerance Test (GTT) in 2019 compared to those with a negative HbA1c and Fasting Glucose in 2020).
