# Supplementary material for: Clinical Outcomes Following a Change in Gestational Diabetes Mellitus Diagnostic Criteria Due to the COVID-19 Pandemic: A Case-Control Study
Source: Int J Environ Res Public Health. 2022 Feb 8;19(3):1884. doi: 10.3390/ijerph19031884 (PMC8835277; doi:10.3390/ijerph19031884)
Supplement: Supplementary file 1 [file ijerph-19-01884-s001.zip › ijerph-1546952-supplementary.pdf]

Table S1. Maternal and neonatal outcomes compared based on those with a positive Glucose Challenge Test (GCT) in 2019 to those with a positive GCT in 2020

	All 2019 n=218	All 2020 n=210	p value
BMI, mean (SD)	26.7 (5.9)	27.3 (6)	0.288
Age in years, mean (SD)	36 (5)	35 (5.1)	0.212
Gestational Age at GCT (weeks), median (SD)	28 (4.6)	28 (4.3)	0.6
Booking weight mean in kg (SD)	72.7 (16.3)	72.9 (16.4)	0.554
Infant head circumference centile, mean (SD)	70.3 (27.6)	75 (28)	0.457
Infant length centile (SD)	74.8 (28.4)	75 (30)	0.926
Birth Weight in g (median, SD)	3633 (505)	3648 (584)	0.688
PPH (EBL >500ml as % of births), n(%)	46 (21.1%)	56 (26.7%)	0.194
Birth weight (>90th centile by gestation and gender)	43 (19.7%)	55 (26.2%)	0.117
Induction of labour (as % of births)	64 (29.4%)	79 (38%)	0.217
Mode of Birth			
Spontaneous Vaginal Birth	110 (50.5%)	101 (48.1%)	0.625
Instrumental Birth	24 (11%)	25 (11.9%)	0.771
Caesarean Section	84 (38.5%)	84 (40%)	0.756
NICU admission	57 (26.3%)	50 (23.8%)	0.558

GA- Gestational Age, GCT- Glucose Challenge Test, NICU-Neonatal Intensive Care Admission, SD-Standard Deviation

Table S2. Comparing all those with a positive Glucose Challenge Test (GCT) but negative second step diagnostic test (Glucose Tolerance Test (GTT) in 2019 compared to those with a negative HbA1c and Fasting Glucose in 2020)

	All GCT positive GDM negative 2019 n=131	All GCT positive but GDM negative 2020 n=167	p value
BMI (mean, SD)	25.4 (6.1)	26.9 (5.7)	0.201
Age (median in years, SD)	36 (5)	36 (4.9)	0.491
Gestational age (weeks) at Glucose Challenge Test (GCT), SD	28 (4.8)	28 (4.1)	0.778
Booking weight in kg (median, SD)	70.1(16.8)	72.2 (16)	0.340
Infant head circumference centile (mean, SD)	71.8 (26.3)	75 (26.2)	0.407
Infant length centile (mean, SD)	75 (30)	79.1 (28.8)	0.463
Birth Weight in grams (mean, SD)	3650 (525)	3680 (570)	0.597
Postpartum haemorrhage (PPH) (EBL >500ml), n(%)	26 (21.1%)	51 (30.5%)	0.08
Birth weight >90th centile	25 (20.3%)	46 (27.5%)	0.158
Gestational Age at birth (weeks), mean, SD)	39.7 (1.5)	39.7 (1.9)	0.7
Induction of labour	32 (26%)	58 (34.7%)	0.324
Mode of Birth			0.560
Spontaneous Vaginal Birth	62 (50.4%)	81 (48.5%)	0.749
Instrumental Birth	11 (8.9%)	22 (13.2%)	0.262
Caesarean Section	50 (40.7%)	65 (39%)	0.718

GA- Gestational Age, GCT- Glucose Challenge Test
